# Soundscape Evaluation Outside a Taoist Temple: A Case Study of Laojundong Temple in Chongqing, China

**DOI:** 10.3390/ijerph19084571

**Published:** 2022-04-11

**Authors:** Hui Xie, Zhaohui Peng, Jian Kang, Chang Liu, Huifei Wu

**Affiliations:** 1 Faculty of Architecture and Urban Planning, Chongqing University, Chongqing 400045, China; pengzhaohui2022@126.com (Z.P.); lc87@cqu.edu.cn (C.L.); wuhf19@163.com (H.W.); 2 Key Laboratory of New Technology for Construction of Cities in Mountain Area, Ministry of Education, Chongqing University, Chongqing 400045, China; 3 Institute for Environmental Design and Engineering, The Bartlett, University College London-14 Upper Woburn Place, London WC1H 0NN, UK; j.kang@ucl.ac.uk

**Keywords:** Taoism, religious spaces, soundscape evaluation, audio-visual interaction

## Abstract

The unique architectural form and religious background of Taoist buildings can lead to a special acoustic environment, but there is a lack of research on the soundscape evaluation of Taoist buildings. Laojundong Taoist Temple was selected as the research site. The psychological and physiological responses of Taoist priests and ordinary people, and strategies for soundscape renovation were investigated by conducting field measurements, interviews, soundwalks, and audio–visual experiments. There was significant negative linear regression between the L_Aeq,5min_ and soundscape comfort (*p* < 0.01). The visual landscape comfort of ordinary people was notably correlated with landscape diversity (*p* < 0.01), whereas their soundscape comfort was markedly correlated with the degree of natural soundscape and audio–visual harmony (*p* < 0.01). The soundscape evaluation by Taoist priests was affected by their belief, activity types, social factors, and spatial positions. With the increasing proportion of the natural elements in the visual landscape in the temple, the acoustic comfort of Taoist priests and ordinary people significantly increased with the addition of bird sounds (*p* < 0.01). However, with the increasing proportion of Taoist scenes, Taoist music only significantly improved the acoustic comfort and heart rate of ordinary people (*p* < 0.01).

## 1. Introduction

Taoism originated in China over 1800 years ago. A global religious census showed that there are nearly 90 million Taoist believers around the world [1]. The Taoist temple is an essential religious place for practitioners to worship, preach, and practice, as well as a space for ordinary people to relax and visit. Influenced by the belief of conforming to nature, the space of the temple is closely connected to mountain terrains and forests, which provide abundant landscapes with natural elements in each hall [2]. Previous research on the acoustic environments of different religious places, such as Taoist temples, Buddhist temples, churches, and mosques, has focused on analysing the impact of architectural components, envelope materials, and sound sources on the reverberation time (T_30_), sound distribution, and other indices of religious space through in situ measurements and simulations. In Buddhist temples and churches, higher courtyard walls of the temple and ceilings of the church enhanced sound reflection and sound field uniformity [3,4,5]. For mosques, the recommended T_30_ for satisfying the needs of all acoustic scenes was greater than 1.0 s [6].

Landscape is the perceived area by people, whose character is due to the action and interaction of natural and/or human factors, while soundscape is defined as the acoustic environment that is perceived and/or understood by a person or people in context, according to ISO 12913-1:2014 [7,8]. Landscape compositions such as vegetations have significant effects on the perceived understanding of the acoustic environment [9,10,11]. Researchers have analyzed the cognitive characteristics and influencing factors of people’s perception of religious environments through questionnaires and interviews. Zhang et al. found that the sound of bells and flowing water enhanced the Buddhist atmosphere, and people were relaxed after experiencing a combination of natural sounds and landscape [12,13]. Jeon et al. revealed that the soundscape of the church did not enhance a religious atmosphere as well as in the Buddhist temple [14]. However, people’s familiarity with the sound of church bells improved their engagement in religious activities [15,16]. In mosques, people were more likely to be immersed in religious activities when hearing religious music [17]. As a qualitative method, grounded theory has been also adopted to several soundscape studies, for instance, in the context of open plan offices and historical museums [18,19,20], but there is no research yet applying ground theory to investigate the soundscape of religious spaces. 

On the other hand, the impact of audio–visual scenes with different characteristics on human physiological responses has gradually received attention. The combination of natural scenes and sounds significantly reduced subjects’ heart rate, heart rate variability, and depth of respiration, whereas unpleasant scenes increased their electromyography activity [21,22,23]. A pleasant urban soundscape enhanced the degree of connection between brain neurons [24]. Moreover, the skin conductivity levels (SCLs) of subjects recovered more quickly in a natural soundscape than in a noisy environment [25]. This shows that natural audio–visual scenes help to relieve people’s stress. Moreover, findings also indicated that the soundscape in the places of religious worship, as an important component of creating a sacred atmosphere, might be associated with desirable mental health outcomes. The frequency of listening to religious music was linked with an increase in life satisfaction and a sense of control, as well as a decrease in death anxiety [26]. The preferences for typical sounds in Buddhist temples, such as bells, wind chimes, and chanting sounds, were significantly correlated with mental health of believers and tourists [27].

Research on soundscape preservation has mainly analyzed the relationships between space, cultural backgrounds, and soundscapes before proposing strategies. The propagation characteristics of the sound field in mosques and churches were studied through digital simulations before proposing restorative methods for sound sources and inner wall materials [28,29]. For urban soundscape protection, five preservation values, namely ecological, comfortable, affective, identifiable, and practical value had been taken into consideration [30]. In the preservation of a historical landscape, the perception of the cultural background, sound events, and urban space form of Lhasa and Dong minority towns in Guizhou, China, were discussed in detail [31,32]. However, the soundscape evaluations in Taoist temples and the corresponding strategies for preserving Taoist soundscape have not been studied yet. This paper aims to investigate the following issues: (1) the characteristics of the acoustic environment in Taoist temples, and (2) the soundscape evaluation, physiological response of Taoist priests and ordinary people towards the soundscape in Taoist temples. Firstly, 24-h sound pressure levels were measured in the Taoist temple and its surrounding road. Then, in-depth interviews and a soundwalk were conducted to obtain the soundscape evaluation of Taoist priests and ordinary people for the Taoist temple, respectively. Thirdly, differences in the physiological response and acoustic comfort between Taoist priests and ordinary people were compared based on audio–visual interaction experiments. Finally, strategies for preserving the Taoist soundscape were formulated.

## 2. Materials and Methods

### 2.1. Case Study Area

**Figure 1:**
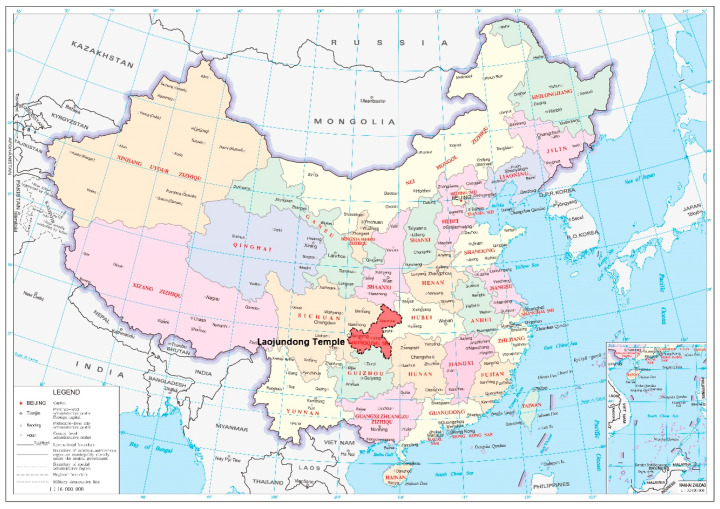
The location of the Laojundong Temple in Chongqing, China.

**Figure 2:**
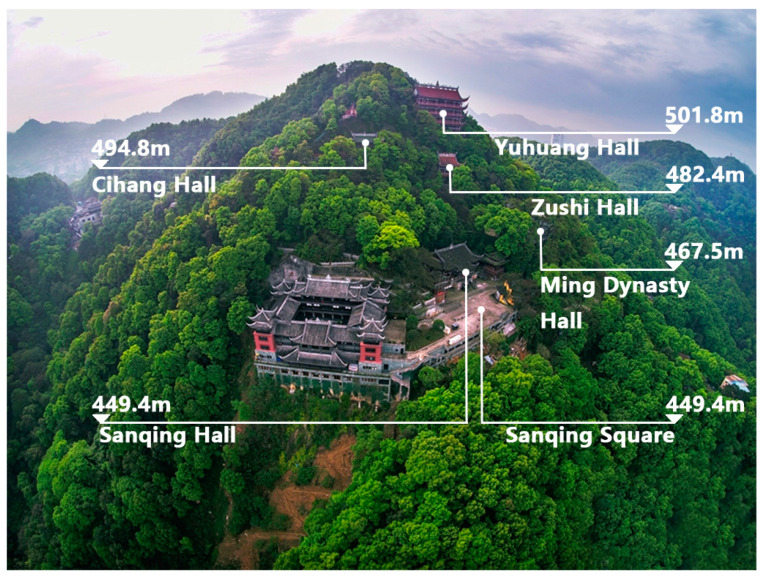
Spatial distribution and elevations of the main attractions of Laojundong Temple.

With more than 1300 years of history, Laojundong Taoist Temple covers an area of over 200,000 m^2^ with several halls on the South Mountain, Chongqing, China, along with rich natural landscape and soundscape resources (Figure 1). The main halls of the Temple are distributed along the contour topography of the mountain and are arranged in sequence with typical Taoist halls such as the Nanshan Gate, Sanqing Hall, and Yuhuang Hall (Figure 2). The altitude difference throughout the whole temple is about 52.4 m. There are approximately 60 Taoist priests living in the temple and thousands of visitors every day. It adopts the classical palace-style structures of traditional Taoist temples, and the inner courtyards inside the temple are integrated with the mountain to form space with different degrees of enclosure [33]. Therefore, Laojundong Taoist Temple appropriately embodies the traditional Taoist temple’s design characteristics of advocating nature and adapting to local conditions and has a significant representative of other Taoist temples.

The temple is affected by notable traffic noise from the urban expressway on its west side and occasional aircraft noise due to the airport on its far north side at the distance of 20 km, which has influenced the daily practice and rest of Taoist priests and caused people to have negative experiences. The square in front of Sanqing Hall has an elevation of 449.4 m, and a vertical distance of 139.1 m from the expressway. The horizontal distance between the edge of the square and the expressway is 244.5 m. The highest point of the temple is Yuhuang Hall, with the elevation of 501.8 m.

### 2.2. Noise Level Measurements

**Figure 3:**
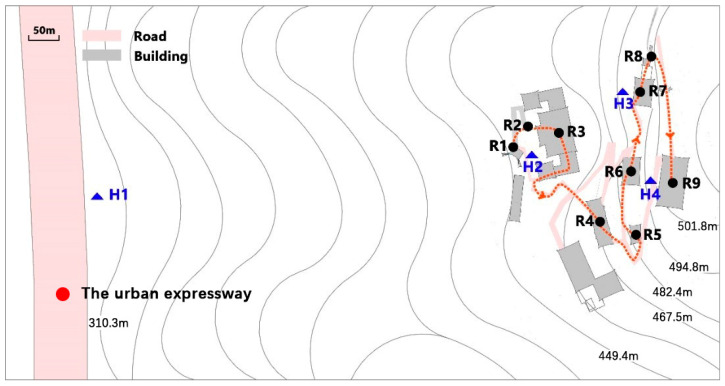
The layout of 24-h sound level measurement points including H1: The urban expressway, H2: Sanqing Square, H3: Cihang Square, H4: Yuhuang Square; soundwalk points including R1: Front Gate, R2: Sanqing Square, R3: Sanqing Hall, R4: Ming Dynasty Hall, R5: Painting Corridor, R6: Zushi Hall, R7: Cihang Hall, R8: Qizhen Hall, R9: Yuhuang Hall.

The in situ measurement was more appropriate than a noise mapping approach to explore the features of the acoustic environment of Laojundong Temple considering the complex mountainous terrain and architectural forms of case study site. As shown in Figure 3, in order to investigate the impact of traffic noise on the Taoist priests and ordinary people in Laojundong Temple, four measuring points (H1–H4) were selected to simultaneously conduct 24-h continuous sound level measurements using a Type I sound meter on 17 January 2019. H1 was chosen on the sidewalk beside the busy expressway at a distance of more than 50 m from the intersection. Other measuring points (H2–H4) were set on the squares in front of the Sanqing, Cihang and Yuhuang Hall where people often stay for religious ceremonies and activities, and were 1.5 m from the surrounding reflective surfaces. When performing the measurement, the temperature was 12 °C, the wind speed was 3.3 m/s, and the humidity was 72%, which was favorable for the propagation of noise in the road-to-temple direction. The average number of vehicles on the urban expressway was 11,927 per hour during the daytime and 2026 per hour at night. The speed limit was 100 km/h. The SPL was recorded at each second in the range of 24 h. 

### 2.3. Soundwalk

A soundwalk was conducted to explore the perceived experiences of ordinary people who weren’t familiar with the Taoist temple. Figure 3 shows the soundwalk route and 9 measuring points that were selected to represent the audio–visual characteristics of Laojundong Temple. R1 is Nanshan Gate, the entrance of the temple. R2 is the square near the Sanqing Hall. R3~R9 are Sanqing Hall, Ming Dynasty Hall, the Painting corridor, Zushi Hall, Cihang Hall, Qizhen Hall, and Yuhuang Hall, respectively. The soundwalk was performed after 2 p.m. on a sunny day (15 °C, humidity 75%, wind speed <5 m/s, favorable for the propagation of noise in the road-to-temple direction), but a different time from the above noise measurements. The weather condition was favourable to ensure the walk comfortability. 

A total of 26 subjects, 12 males and 14 females with normal hearing condition and fitness were randomly recruited from Chongqing University and participated in the soundwalk. To ensure the accuracy of the evaluation, all participants were trained to ensure their familiarity with each evaluation question before starting. During the soundwalk, the participants were asked to remain at each measuring point for 5 min before their evaluation and stay quiet all the time. At each measuring point, a Type I sound meter was used for a 5-min SPL test, and the L_Aeq,5min_ was calculated as the acoustic indicator accordingly. A digital recorder (Sony PCM-D100) was used to take a 5-min ambient recording.

In accordance with ISO/TS 12913-2:2018, the questionnaire was constructed on the basis of a previous study [34], and consisted of evaluations of the soundscape and landscape in Laojundong Taoist Temple. The aim of the first part was to describe the soundmark, sound preference, and sound expectation in the current acoustic environment. In the second part, the soundscape and visual landscape was evaluated through eight adjectives describing their attributes: enclosed/open, artificial/natural, weak/strong, disharmonious/harmonious, and uncomfortable/comfortable, which were rated on a nine-point category scale to assess the degree of spatial enclosure, the degree of natural soundscape, soundscape/landscape diversity, audio–visual harmonies, and soundscape/landscape comfort, respectively.

### 2.4. In-Depth Interview

Different from normal people, Taoist priests were quite familiar with the surrounding environment of the temple. In order to further learn how Taoist priests evaluate the acoustic environment of the temple, 11 Taoist priests (male-to-female = 5:6) were randomly selected for in-depth interviews, and the results were qualitatively analyzed using the grounded theory. Semi-structured questions were adopted to collect the interview data of Taoist priests, aiming to obtain more content associated with participants’ perception of sound sources, their sound expectation, and their overall satisfaction.

Each Taoist priest was interviewed for an average of 20 min in a private, quiet room in the Temple. To ensure that the interviewees were familiar with the acoustic environment of the Temple, only Taoist priests with more than one year of practice were approached. The semi-structured questions included:

1. What types of sounds do you perceive in Laojundong Taoist Temple?
2. How does the acoustic environment of the Temple affect your daily activities and rest?
3. What is your overall evaluation of the acoustic environment of the Taoist temple?
4. What kind of sounds do you expect to hear during your daily activities and rest?

During the interview, important events that related to the perceived acoustic environment and recurrent topics that were expressed by participants were identified and added as new questions. Once no more additional data could be obtained to further develop the properties of the category regarding the soundscape evaluation, the interviews were stopped as theoretical saturation was reached [35].

There were three stages, namely, open coding, axial coding, and selective coding, that were then carried out to process the interview materials [28]. In the open coding, the materials were separated into several keywords. Then, in the axial coding, the relationship between each keyword was analyzed and the same words were grouped together to generate categories. Each category was titled to show its content. Finally, in the selective coding, the connections between the different category were explored, and a possible explanation emerged to describe the cognitive characteristics of Taoists in response to the acoustic environment.

### 2.5. Audio-Visual Experiment

#### 2.5.1. Visual and Auditory Materials

**Figure 4:**
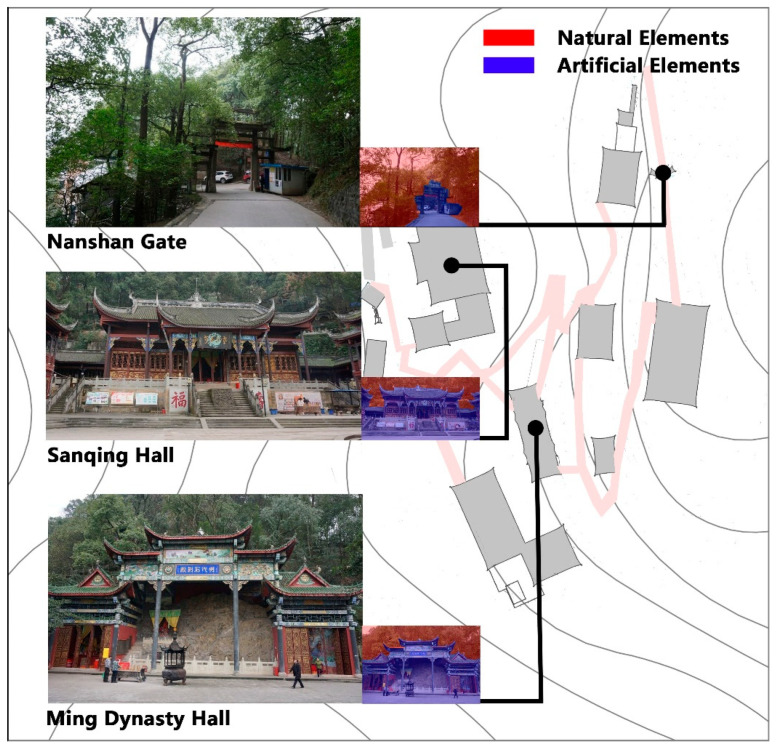
Three visual materials representing typical landscape characteristics of Laojundong Temple.

The audio-visual experiment was carried out to investigate the differences of the acoustic evaluation between Taoists priests and ordinary people. According to the preliminary survey of the visual landscape in Laojundong Taoist Temple, visual materials were obtained by taking photographs at three typical sites representing the characteristics of visual landscape elements: Nanshan Gate, which has a largest proportion of natural landscape; Ming Dynasty Hall, which has a balanced proportion of natural landscape and Taoist Hall; and Sanqing Hall, which has the largest proportion of Taoist architecture (Figure 4). All the visual materials were recorded from 10:00 a.m. to 11:00 a.m. on the same day, and pictures were taken at a horizontal eye level (1.5 m above the ground) using a Canon 7D camera. 

**Figure 5:**
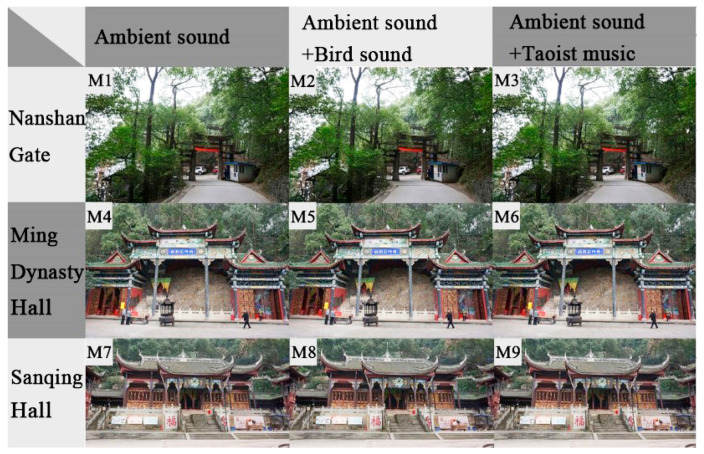
9 combinations (M1–M9) of three visual materials and sounds representing the typical characteristics of landscape and soundscape in Laojundong Temple.

The auditory materials included ambient sounds, bird sounds, and Taoist music. The ambient sounds were recorded in Ming Dynasty Hall at the elevation of 1.5 m and a distance of more than 1.5m from other reflecting surfaces. The wind speed was less than 5 m/s when recording, and a Type 1 sound level meter was used to measure the SPL at the same time. The background sound in Ming Dynasty Hall was the traffic noise from the urban expressway, and the foreground sound was the sound of birds and religious instruments. Adobe Audition software was used to extract 75 s of audio files as the ambient sound material in the experimental process. These three kinds of sound materials—ambient sounds, bird sounds, and Taoist music—were arranged and combined with the three of the above visual scenes to form a combination of nine audio–visual materials, as illustrated in Figure 5. There were three audio-visual scenes with only ambient sound (M1, M4, M7) that were set as the control groups.

#### 2.5.2. Procedure

The experimental participants were 20 Taoist priests (male-to-female ratio = 9:11) and 20 university students (male-to-female ratio = 9:11) with normal vision and hearing conditions, who were different from the participants in the previous soundwalk and in-depth interview. The experiment was conducted in a quiet testing room without any disruptions. The visual reproduction equipment was a 19-inch television display, the auditory reproduction equipment was a two-channel speaker, and the physiological indicator instrument was a portable and wearable monitoring device. The participants were asked to look at the visual materials and listen to the sounds. The audio–visual materials were played at random, and each kind of audio–visual material was continuously played for 75 s. The interval between each play was 35 s, during which the heart rate of the subjects was recorded by the monitoring device. The subjects scored their acoustic comfort evaluation according to their listening experience while viewing the images. An 11-level semantic scale was adopted, and a pair of adjectives with opposite meanings, namely, ‘very uncomfortable’ and ‘very comfortable’, was used. The study was conducted in accordance with the Declaration of Helsinki, and informed consent of each participant was obtained prior to the conducting of the experiment.

### 2.6. Statistic Analysis

SPSS 22.0 was used to calculate each index that was included in the questionnaire. The Kolmogorov–Smirnov test and Spearman test were performed to analyze the normal distribution of each parameter and the correlation between the soundscape/visual components, soundscape/visual landscape comfort, and L_Aeq,5min_, respectively, and the regression analysis was used to further investigate how L_Aeq,5min_ was associated with the soundscape comfort. Furthermore, the Kruskal–Wallis test and ANOVA for repeated measurements were used to analyze the differences between the audio–visual combinations and control groups in terms of acoustic comfort and heart rate variations of Taoist priests and ordinary people, respectively.

## 3. Results

### 3.1. Sound Sources and Measurement Results

**Figure 6:**
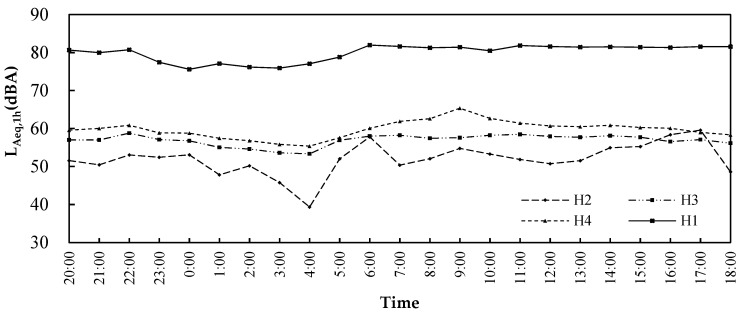
L_Aeq,1h_ at four measuring points per hour.

Since sound level measurements at H1~H4 were performed simultaneously, Figure 6 compares the time variations of acoustic environment at four measuring points in 24 h. It can be seen that the average L_Aeq,1h_ of H2 (Sanqing Hall), H3 (Cihang Hall), and H4 (Yuhuang Hall) in 24 h were between 39.4 and 65.4 dBA, and the traffic noise of H1 (along the expressway) reached a maximum L_Aeq,1h_ of 82.0 dBA. Compared with the other two halls, the similarities between fluctuations of the L_Aeq,1h_ at Yuhuang Hall and the traffic noise along the expressway were the highest, and the L_Aeq,1h_ of Yuhuang Hall was the highest among them, indicating that Yuhuang Hall was the most seriously affected by traffic noise. The L_Aeq,1h_ in front of Sanqing Hall fluctuated the most dramatically among the four measuring points. From 5:00 p.m. to 4:00 a.m., the L_Aeq,1h_ gradually decreased from 59.6 dBA to 39.4 dBA with the dwindling number of tourists and the end of the Taoists priests’ daytime activities. Conversely, the L_Aeq,1h_ gradually increased as the Taoist priests played their religious instruments, chanted, and performed other spiritual activities at 4:00 a.m. The maximum L_Aeq,1h_ of 57.8 dBA was reached at 6:00 a.m., when the priests hit the bells and drums on both sides of Sanqing Hall. However, the L_Aeq,1h_ remained relatively steady in Cihang Hall, which might be related to the relatively low crowd density at the temple.

According to the result of L_Aeq,5min_ at each point of the soundwalk, R1 and R2 were dominated by traffic noise, mahjong (a tile-based popular game in China) sounds, and crowd noise at 52.0 dBA and 55.7 dBA, respectively. L_Aeq,5min_ at R4 and R5 with rich natural landscape elements was at 51.4 dBA and 53.1 dBA, where the main sound sources were natural sounds such as the sound of birds, wind, and insects. R3 and R6, where people usually stay for praying, were dominated by crowd and sounds that were related to Taoist activities such as chanting, ritual instruments, Taoist music, and drawing lots, with values at 51.1 dBA and 55.4 dBA. Moreover, R7–R9 were gradually influenced by the traffic noise of the urban expressway, with L_Aeq,5min_ reaching the maximum at 62.7 dBA.

### 3.2. Temple Soundscape Evaluation 

#### 3.2.1. Soundwalk Results

**Figure 7:**
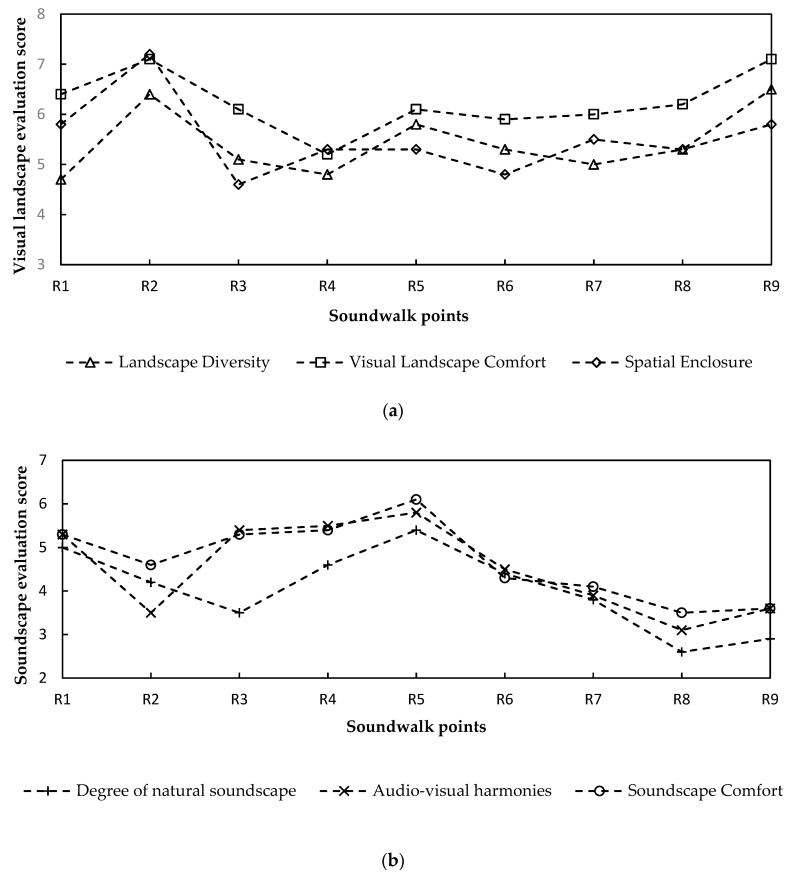
The score of soundscape evaluation at each soundwalk point: (**a**) visual landscape comfort evaluation and (**b**) soundscape comfort evaluation.

As shown in Figure 7, among all the evaluation scores at each soundwalk point, only the mean visual landscape comfort score exceeded 5, suggesting that the participants regarded the visual landscape in Taoist temples as comfortable. Among all the measuring points, R2 (Sanqing Square) enjoyed the highest landscape diversity, spatial enclosure, and visual landscape comfort, which might be related to the open and diverse visual space of the square. From R2 to R5 (Painting corridor), the visual landscape comfort score of the participants decreased by 14% with the decrease in the landscape diversity, whereas the soundscape comfort score increased by 33% with the increase in the score of audio–visual harmonies. From R5 to R9 (Yuhuang Hall), the visual landscape comfort score increased by 16% with the increase in spatial enclosure and landscape diversity. However, the soundscape comfort score decreased by 41% with the decline in the degree of natural soundscape, and the score of audio–visual harmonies. The audio–visual harmonies, the degree of natural soundscape, and the soundscape comfort of the participants was the highest at R5. This shows that the needs of ordinary people in the Taoist temple for landscape and soundscape comfort were different. Landscape with natural elements and natural soundscape were more likely to improve ordinary people’s soundscape comfort.

Furthermore, according to the analysis of the sound preference of the participants, natural sounds such as bird and wind sounds were preferred by people, whereas mahjong, traffic noise, and other artificial sounds that were irrelevant with Taoism made them feel bored. As a result, 42.4% of the participants expected to reduce the impact of the traffic noise and 20.9% of them looked forward to hearing more natural sounds around the Temple.

#### 3.2.2. Factors Influencing Soundscape Evaluation

**Figure 8:**
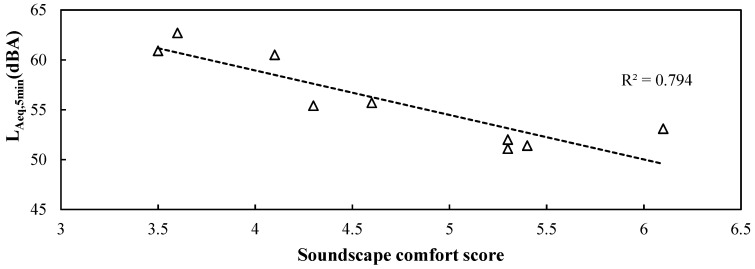
The relationship between the L_Aeq,5min_ and the soundscape comfort score at each soundwalk point.

As shown in Table 1, the soundscape comfort was correlated with audio–visual harmonies, degree of natural soundscape, and soundscape diversity, with correlation coefficients ranging from 0.716 to 0.918. On the other hand, visual landscape comfort was correlated with landscape diversity and spatial enclosure. Higher scores of audio–visual harmonies and degree of natural soundscape significantly improved the soundscape comfort score (*p* < 0.01). This implies that the higher the proportion of natural soundscape and harmonies between landscape and soundscape that is perceived by people in the Taoist temple, the better the evaluation of soundscape comfort scored by people. On the other hand, the visual landscape comfort score was significantly correlated with landscape diversity (*p* < 0.01), indicating that the abundant visual elements in Taoist temples could effectively improve the visual landscape comfort of ordinary people. In addition, statistically significant correlations between soundscape comfort and soundscape diversity; and between visual landscape comfort and spatial enclosure were found (*p* < 0.05). This indicates that open space and diverse sounds could also improve the landscape/soundscape comfort of ordinary people. Furthermore, regression analysis showed a significant negative linear regression between the L_Aeq,5min_ and soundscape comfort score (R^2^ = −0.794, *p* < 0.01). As shown in Figure 8, when the L_Aeq,5min_ surpassed 55 dBA, the soundscape comfort score was rated less than 5. This means that the acoustic environment makes ordinary people feel uncomfortable when the L_Aeq,5min_ is over 55 dBA.

### 3.3. Charecteristic of Soundscape Evaluation of Taoist Priests

#### 3.3.1. Noise Sources

Although the influence of aircraft noise was significantly reduced due to the 20 km distance between the airport and the Laojundong Temple, some Taoist priests still felt disturbed by the low-frequency noise at night. Taoist priests thought that noises were mainly from the urban expressway and aircraft. Most of them complained that those noises could be heard during the day and at night, and they were more obvious at night. The mobility and individual occurrence of aircraft noise made Taoist priests believe that the noise was the loudest when the distance between aircraft and their position was the smallest. In addition, they felt that traffic noise was louder than other sounds from 11:00 p.m. to 1:00 a.m. Some Taoist priests thought that the impact of traffic noise on foggy days was relatively insignificant, which might be related to the low traffic flow and low speed of vehicles on foggy days. On the other hand, noises had a serious impact on their quality of sleep, which not only prolonged their time to fall asleep but also lowered their sleep quality. 

#### 3.3.2. Social Context 

There are many factors affecting Taoist priests’ sensitivity and annoyance to noise, such as their practice time, age, physical health, habituation to noise, and understanding of Taoist belief, which is consistent with previous results [36]. With the increase in their practice time, their sensitivity and annoyance to noises decreased, and their habituation to noise improved. Some Taoist priests with long practice times were less sensitive to noise and thought it was not very noisy. Noise had a greater impact on the sleep quality of an older Taoist priest with poor health.

#### 3.3.3. Spatial Distribution

Many environmental factors affected Taoist priests’ annoyance to the noise, including areas with different elevations, spatial positions, and crowd densities. They thought that Yuhuang Hall, the highest architecture of Laojundong, was most seriously affected by traffic and aircraft noise, whereas Sanqing Hall, with the lowest elevation, was less influenced by traffic noise. The side of the Taoist Halls facing the urban expressway was more affected by noises than that facing away from it, which is related to the Taoist Hall’s obstruction to noises. Some of them believed that the relatively remote places of Taoist temples were quieter, such as the camphor forest, where there were fewer tourists.

#### 3.3.4. Activity Types

The behavioral factors affecting their perception of noise included passive noise reduction behavior and residence time. Taoist priests adopted various measures to reduce their annoyance to the noise, such as diverting attention, plugging their ears with cotton, or masking it with the sound of a television. Some of them believed that the longer that they stayed in the same position in the temple, the worse the perceived noise would be.

The daily activities of Taoist priests included religious rites, practice, and rest. As for sound expectations, they believed that the sounds of insects and birds were helpful to their practice and made them feel comfortable, but the sound of water was too noisy when they were practicing. However, some of them had higher requirements for the environment when they practiced and rested, and expected to hear as few sounds as possible. For Taoist ritual activities, the Taoist sounds such as Taoist music, chanting, and ritual sutras not only had special Taoist meaning but also created the atmosphere of Taoism.

#### 3.3.5. Taoist Belief 

Influenced by the Taoist concept of harmony between humans and nature, Taoist priests preferred natural sounds, such as bird, wind, and rain sounds. Artificial sounds such as Taoist music, bell and drum sounds, and chanting were regarded as harmonious sounds of nature with a high comfort level. Moreover, they believed that the general acoustic environment always contained different kinds of sound sources, including noises, so they had an inclusive and accepting attitude towards noises, which helped to reduce their annoyance to them.

#### 3.3.6. Strategies for Improving Acoustic Environment

With regard to noise reduction strategies, Taoist priests tended to use acoustic ventilation windows that were less damaging to the landscape with natural elements than constructing noise barriers. They also thought that adding unnatural sounds would not make them feel calm, so the sounds of rain, insects, and birds improved the sound environment better than Taoist music. In addition, they stated that the above sounds should only be played in the daytime, as playing these sounds at night would affect their sleep quality.

### 3.4. Audio-Visual Interaction Results

#### 3.4.1. Psychological and Physiological Responses of Taoist Priests and Ordinary People

**Figure 9:**
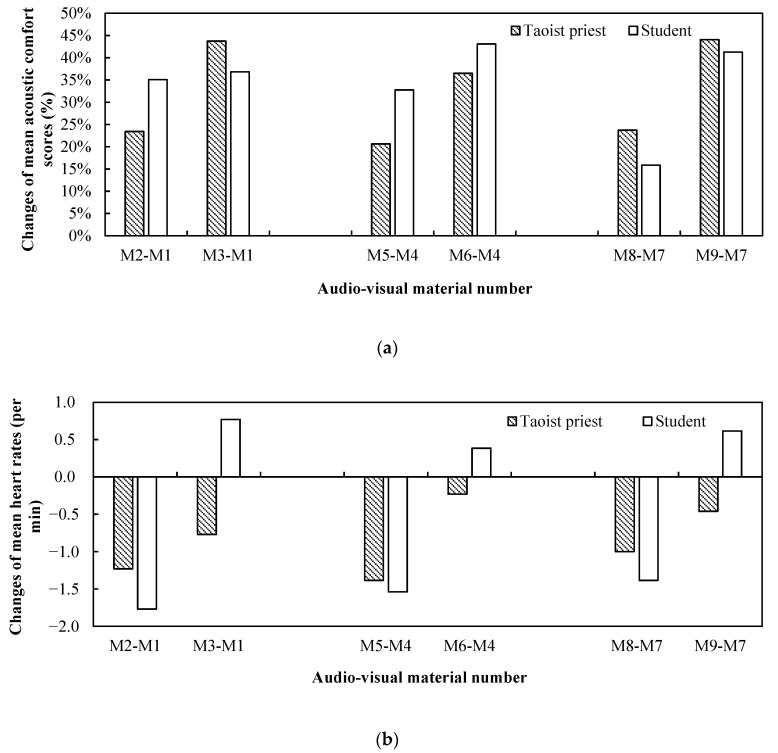
Audio-visual interaction results of Taoist priests and ordinary people after experiencing audio-visual materials: (**a**) changes of the mean acoustic comfort scores after adding bird sound and Taoist music to the control groups; (**b**) changes of the mean heart rates after adding bird sound and Taoist music to the control groups.

Figure 9 shows the changes of both the mean acoustic comfort scores and heart rates of Taoist priests and ordinary people when they experienced different kinds of audio–visual scenes compared to the control groups. Since the acoustic environment of Laojundong Temple was largely affected by the traffic noise, three audio-visual scenes with only ambient sound (M1, M4, M7) were set as control groups to investigate the effects of adding bird sound and Taoist music on the psychological and physiological responses of the participants. 

As expected, regarding the acoustic comfort, both Taoist priests and ordinary people felt uncomfortable when they experienced audio–visual scenes (M1, M4, M7) accompanied only by ambient sound as the control samples. However, the mean acoustic comfort scores of Taoist priests and ordinary people were all above ‘5’ when they experienced audio–visual scenes (M2, M3, M5, M6, M8, M9) with the addition of bird sounds or Taoist music to the ambient sound. This indicates that the acoustic comfort of Taoist priests and ordinary people were markedly improved by bird sounds and Taoist music. As illustrated in Figure 9a, for Taoist priests, the improvement of mean acoustic comfort score after adding Taoist music (M3, M6, M9) was greater than that after adding bird sounds (M2, M5, M8). When they experienced the scene of Sanqing Hall with bird sound (M8) and Taoist music (M9), the increasements were both at the maximum of 23.73% and 44.07%, respectively, compared with the other two visual scenes. For the acoustic comfort of students, when experiencing the Nanshan gate after adding bird sound (M2), the maximum improvement of the mean comfort score was 35.09%. This suggested that the combination of rich vegetation and bird sounds in Taoist temples could improve the acoustic comfort of the ordinary people.

Figure 9b demonstrates how the mean heart rates of Taoist priests and students changed compared to the control group. Among the nine kinds of audio–visual scenes, the mean heart rate of both Taoist priests and ordinary people decreased when they experienced three audio–visual scenes (M2, M5, M8) after bird sounds were added to the ambient sound, compared with the controlled audio–visual scenes (M1, M4, M7) with only ambient sound. The heart rate of Taoist priests dropped the most by 1.90%, after adding bird sound into the Ming Dynasty Hall (M4), whereas the heart rate of the students decreased most significantly by 2.49% after bird sound was added to Nanshan Gate (M2). However, after experiencing three visual materials plus Taoist music (M3, M6, M9), the mean heart rate of the students all increased, in contrast to the decreased heart rate of Taoist priests. This might be related with the curiosity and excitement of students about the mysterious Taoist music when entering the temple, but the familiar sound of Taoist music still made priests feel relaxed. 

#### 3.4.2. The Influence of Visual Elements on the Acoustic Comfort and Heart Rate 

Table 2 shows that in the audio–visual scenes containing only ambient noise, there was a statistical difference in the mean heart rate of ordinary people when they saw the visual scenes of Nanshan Gate and Sanqing Hall (* *p* < 0.05). There was no notable difference in other visual scenes. This might be attributable to the green vegetation in the visual scene of Nanshan Gate, which alleviated ordinary people’s pressure that is induced by noise. As the visual scene changed from Sanqing Hall to Nanshan Gate (M8 and M2), the mean heart rate of ordinary people was significantly reduced when watching the audio–visual scene in which the bird sounds were added to ambient sound (** *p* < 0.01). However, as the visual scene changed from Nanshan Gate and Ming Dynasty Hall (M2 and M5), the heart rate of ordinary people statistically differed between that two audio–visual scenes (* *p* < 0.05). This suggests that the higher the proportion of natural scenery in the Taoist temple, the better the restorative effect of bird sounds on ordinary people. Furthermore, when ordinary people experienced the audio–visual scenes from Nanshan Gate to Sanqing Hall (M3 and M9) with ambient sound containing Taoist music, their mean heart rate notably increased (** *p* < 0.01). However, there was no significant difference in the mean heart rate of Taoist priests who experienced the same audio–visual scene. The results demonstrated that Taoist music was more likely to make ordinary people excited in the typical Taoist visual scenes of the temple than in the landscape dominated by natural elements.

#### 3.4.3. The Influence of Sound Types on the Acoustic Comfort and Heart Rate 

As provided in Table 3, when experiencing the audio–visual scenes (M1 and M3; M4 and M6) of Nanshan Gate and Ming Dynasty Hall, the mean acoustic comfort score of Taoist priests and ordinary people statistically differed between before and after Taoist music in the ambient sound, respectively (* *p* < 0.05). When they saw the visual scene of Sanqing Hall after the addition of Taoist music (M7 and M9), the mean acoustic comfort score of ordinary people significantly increased (** *p* < 0.01), but that of the Taoist priests did not. This shows that Taoist music could increase the acoustic comfort of ordinary people for any visual scene. Moreover, the enhancement of the visual landscape with Taoism characteristics could advance the effect of Taoist music on ordinary people’s acoustic comfort. On the contrary, Taoist music did not contribute to Taoist priests’ acoustic comfort in the hall. In addition, when watching the visual scenes of Nanshan Gate and Sanqing Hall with the ambient sound containing either bird sounds or Taoist music (M2 and M3; M8 and M9), ordinary people’s mean heart rate statistically differed between before and after bird sounds or Taoist music (* *p* < 0.05), which was not found in the scene of Ming Dynasty Hall. This indicates that in the visual landscape of the Taoist temple with noticeable natural or Taoist characteristics, bird sounds and Taoist music should not be added together to improve the overall evaluation of the acoustic environment by ordinary people.

## 4. Discussion

### 4.1. Characteristics of the Acoustic Environment of Taoist Temple 

The acoustic environment of Laojundong Temple was clearly affected by the characteristics of the visual landscape, the crowd density, and the time period. The areas with a high proportion of natural vegetation in the visual landscape of the temple provided habitats for birds and insects, and people preferred to stay and rest in these areas. The corresponding dominant sources were therefore natural sounds. While in the visual landscape with a high proportion of Taoist temple, people tended to conduct religious activities such as worship and praying, so the dominant sound sources were generally from Taoism-related activities. On the other hand, the crowd density of Taoist temples was affected by the fatigue of the crowd climbing. With the increase of temple elevation, the crowd density gradually decreased, possibly leading to more human activity sounds in halls with relatively lower elevation. Furthermore, the number of visitors to the main halls of the Taoist temple changed dynamically during the time period from opening to closing during the day, thus the sound level fluctuation at these halls under the impact of the sounds from human-beings was greater than that of halls at relatively high places. In previously studied Buddhist temples, churches, mosques, and other religious places, the plane layouts were intensively distributed, and there was no significant change in their elevation. Therefore, the L_Aeq_ of the religious spaces that were mentioned above was less affected by the terrain [37,38,39].

### 4.2. Religious Perception of Sound Sources Influencing Acoustic Comfort

Compared with ordinary people, Taoist priests’ acoustic comfort were more influenced by the Taoist religious principles, emphasizing harmony between human beings and nature. In terms of traffic noise, some of the Taoist priests were less bothered than ordinary people. This might be due to the influence of Taoist belief: the Taoist priests, considering the traffic noise to be a part of the environment, were more accustomed to and tolerant of it. Compared with traffic noise, bird sounds accorded with both the Taoist priests’ belief and ordinary people’s cognition of the natural environment. Therefore, after adding bird sounds to traffic noise, the acoustic comfort of both was largely elevated. Furthermore, with the increasing types of natural sound sources in the Taoist temple, the soundscape comfort of ordinary people rose accordingly. This is consistent with the results for park soundscapes [40]. In terms of Taoist music, due to their different understanding of Taoism, ordinary people ascribed Taoist music with a typical symbolic meaning, while Taoist priests thought that Taoist music was caused by humans. Therefore, adding Taoist music to traffic noise only diverted ordinary people’s attention from the noise, thus effectively improving their acoustic comfort.

In contrast to the above results, research on the soundscapes of Buddhist temples and churches showed that the sound of streams could create a favorable Buddhist atmosphere, whereas the sound of a bell in churches did not deepen people’s feeling of participation in religion. A possible explanation is that the religious meanings and functions of different sound sources vary among diverse religious places. In the soundscape of Buddhist temples and churches, monks and ordinary people prefer the sound of streams owing to the religious meaning of Buddhist meditation. However, in the church soundscape, sound sources such as the bell and choir, which ordinary people had often heard in their daily life, were so familiar that they were unable to become immersed in the religious atmosphere [41].

### 4.3. The Influence of Visual Landscape with Different Features on Acoustic Comfort

For the Taoist priests and ordinary people that were exposed to the scene with bird sounds and natural landscape, as the proportion of natural landscape increased, their acoustic comfort significantly increased. On the other hand, with the increasing proportion of the Taoist landscape in the audio–visual scene, the acoustic comfort of ordinary people markedly improved after Taoist music was added. However, the acoustic comfort of Taoist priests did not change significantly. This means that Taoist priests had the same expectation of nature as ordinary people except for Taoist music. Moreover, the elevation and position of the visual landscape in Taoist temples had an effect on visual landscape comfort, whose change was inversely related to that of soundscape comfort. From Nanshan Gate to the Painting corridor (R1~R5), the sight was blocked by dense vegetation, leading to lower visual diversity and higher spatial enclosure, except for Sanqing Square (R2), which is relatively open. This resulted in the lower visual landscape comfort of ordinary people in these areas. From the Painting corridor to Ming Dynasty Hall (R5~R9), with the increasing number of platforms, the spatial enclosure and landscape diversity were continually improved, which led to the rising visual landscape comfort of ordinary people. This is inconsistent with the previous study that church space with high spatial enclosure could enhance people’s sense of tranquillity [13]. This might be associated with the surrounding environment where the Taoist Temple has richer natural landscape, whereas the church is generally more adjacent to the urban environment. 

### 4.4. Influence of Audio–Visual Scenes with Different Features on the Heart Rate

In the audio–visual experiment, in response to the combination of ambient sound containing bird sounds and a rich natural landscape, the heart rate of ordinary people decreased significantly. This indicates that bird sounds in the Taoist temple also relieved ordinary people’s tension that was induced by the noise, which is consistent with previous findings [42]. Interestingly, adding bird sounds to the Taoist scene resulted in a notable decrease in the heart rate of Taoist priests, whereas the change in the ordinary people’s heart rate was not notable. A possible explanation is that bird sound comes from natural environment which accords with Taoist belief. However, when the Taoist scene was matched with Taoist music, ordinary people’s heart rate increased significantly. For them, proper excitement helped to increase their sense of religious immersion. However, Taoists’ heart rates did not notably change. This might also be due to the reason that Taoist music is a symbol of Taoism for ordinary people, while Taoist priests are already familiar with that. Although the number of participating subjects were restricted due to the challenges of recruitment, it can still be found that soundscapes or visual landscapes with Taoist characteristics tended to promote physical and mental relaxation. 

### 4.5. Strategies for Improving the Soundscape of the Taoist Temple

As a representative Taoist temple, the strategies for improving the soundscape that was obtained from Laojundong Temple can be reasonably extrapolated to other Taoist temples. Firstly, as suggested by the location of Laojundong Temple, the newly built Taoist temples should fully consider the rich natural soundscape and the visual landscape with Taoist characteristics when selecting the site. Protecting the quality of bird habitats such as vegetation around the Taoist temple will help to improve the diversity and naturalness of the soundscapes. In core areas such as the main halls of the Taoist temple, enhancing the playing frequency of Taoist-related sounds such as Taoist music will help to enhance the immersion of ordinary people. Meanwhile, the courtyards and trail areas that were dug according to the topography of the mountain in Laojundong Taoist Temple also reflected the vital strategies of integrating with the surrounding environment, which formed visual spaces with different degrees of openness, and beneficial to the privacy needs of Taoist priests as well as ordinary people. For the Taoist temple with low landscape openness, its visual landscape comfort can be improved by means of overhead platforms and various paving methods. In addition, according to Taoist priests’ expectations for the acoustic environment, playing natural sounds such as bird sounds and insect sounds that are reproduced by electronic means during practice periods in priest activity areas can reduce Taoist priests’ annoyance to noise.

## 5. Conclusions

Using Laojundong Temple as an example, based on SPL measurements, a soundwalk, in-depth interviews, and an audio-visual experiment, this study investigated the characteristics of the acoustic environment, subjective evaluation, and heart rate features of Taoist priests and ordinary people and developed strategies for the protection of the Taoist soundscape. 

The acoustic environment of the Laojundong Temple was notably affected by the traffic noise. The range of L_Aeq_,_1h_ of the front squares of each hall in 24 h were between 39.4 dBA~65.4 dBA. The soundscape evaluation of the Taoist priests was mainly influenced by their own understanding of Taoist beliefs, while the soundscape evaluation of students was mainly influenced by the diversity, naturalness, and audio-visual harmonies of the audio-visual environment. Regarding the landscape with rich natural elements of the Taoist temple, the sound of birds significantly improved the acoustic comfort of both Taoist priests and students. In contrast, with regard to the landscape with rich Taoist elements, the Taoist music only made ordinary people feel immersed in the context of Taoism. Strategies on improving the soundscape of the Taoist temples should be based on the characteristics of the visual landscape, the expectation of people on soundscape, and their activities, so as to minimize the negative impact of noise on their soundscape evaluation.

In future studies, apart from birds and Taoist music, more common sound sources could be added to explore the influence of varied audio–visual scenes on physiological indices, considering Taoist temples featured with other characteristics of acoustic environment. 

## Figures and Tables

**Table 1:** Spearman’s correlation coefficient between the soundscape/landscape elements and subjective comfort levels at Laojundong Temple. Significant correlations are marked with (* *p* < 0.05) and (** *p* < 0.01).

Categories	SE	VLC	LD	VLT	SC	SD	DNS	AVH
SE	1							
VLC	0.668 *	1						
LD	0.630	0.806 **	1					
VLT	0.292	0.288	−0.104	1				
SC	−0.120	−0.374	−0.249	0.164	1			
SD	−0.345	−0.158	−0.256	0.543	0.716 *	1		
DNS	0.054	−0.330	−0.214	0.452	0.831 **	0.703 *	1	
AVH	−0.425	−0.540	−0.458	0.097	0.918 **	0.741 *	0.748 *	1

SE: Spatial enclosure, VLC: Visual landscape comfort, LD: landscape diversity, VLT: Visual landscape type, SC: Soundscape comfort, SD: Soundscape diversity, DNS: Degree of natural soundscape, AVH: Audio-visual harmonies.

**Table 2:** Significant differences for acoustic comfort and heart rate of Taoist priests and ordinary people between two different scenes of Laojundong temple under the same sound, emerging from Kruskal–Wallis test and ANOVA for repeated measurement test, respectively (* *p* < 0.05, ** *p* < 0.01) (AS: Ambient sound, BI: Bird sound, TA: Taoist music).

		AS			AS + BI			AS + TA		
		M1 and M4	M1 and M7	M4 and M7	M2 and M5	M2 and M8	M5 and M8	M3 and M6	M3 and M9	M6 and M9
Acoustic comfort	Taoist priest	0.775	0.392	0.273	0.836	0.679	0.835	0.601	0.542	0.930
	Student	0.671	0.355	0.673	0.895	0.409	0.753	0.346	0.115	0.367
Heart rate	Taoist priest	0.864	0.893	0.771	0.809	0.802	0.405	0.684	0.702	0.954
	Student	0.186	0.041 *	0.547	0.011 *	0.000 **	0.191	0.181	0.006 **	0.357

**Table 3:** Significant differences for acoustic comfort and heart rate of Taoist priests and ordinary people between two different sounds under the same scene of Laojundong temple, emerging from Kruskal–Wallis test and ANOVA for repeated measurement test, respectively (* *p* < 0.05, ** *p* < 0.01).

		Nanshan Gate			Ming Dynasty Hall			Sanqing Hall		
		M1 and M2	M1 and M3	M2 and M3	M4 and M5	M4 and M6	M5 and M6	M7 and M8	M7 and M9	M8 and M9
Acoustic comfort	Taoist priest	0.338	0.036 *	0.311	0.213	0.022 *	0.735	0.329	0.075	0.402
	Student	0.035 *	0.049 *	1.000	0.137	0.011 *	1.000	0.847	0.004 **	0.105
Heart rate	Taoist priest	0.344	0.366	0.747	0.105	0.804	0.226	0.018 *	0.705	0.641
	Student	0.073	0.421	0.023 *	0.461	0.808	0.211	0.371	0.065	0.042 *

## Data Availability

The datasets generated during the current study are available from the corresponding author on reasonable request.
